# Dialogues with large language models reduce conspiracy beliefs even when the AI is perceived as human

**DOI:** 10.1093/pnasnexus/pgaf325

**Published:** 2025-10-14

**Authors:** Esther Boissin, Thomas H Costello, Daniel Spinoza-Martín, David G Rand, Gordon Pennycook

**Affiliations:** Department of Psychology, Cornell University, Ithaca, NY 14853, USA; Department of Psychology, American University, Washington, DC 20016, USA; Department of Psychology, Cornell University, Ithaca, NY 14853, USA; Sloan School of Management, Massachusetts Institute of Technology, Cambridge, MA 02142, USA; Institute for Data, Systems, and Society, Massachusetts Institute of Technology, Cambridge, MA 02142, USA; Department of Brain and Cognitive Sciences, Massachusetts Institute of Technology, Cambridge, MA 02142, USA; Department of Psychology, Cornell University, Ithaca, NY 14853, USA; Hill/Levene Schools of Business, University of Regina, Regina, Saskatchewan, Canada S4S 0A2

**Keywords:** debunking, artificial intelligence, belief change, conspiracy beliefs

## Abstract

Although conspiracy beliefs are often viewed as resistant to correction, recent evidence shows that personalized, fact-based dialogues with a large language model (LLM) can reduce them. Is this effect driven by the debunking facts and evidence, or does it rely on the messenger being an AI? In other words, would the same message be equally effective if delivered by a human? To answer this question, we conducted a preregistered experiment (*N* = 955) in which participants reported either a conspiracy belief or a nonconspiratorial but epistemically unwarranted belief and interacted with a LLM that argued against that belief using facts and evidence. We randomized whether the debunking LLM was characterized as an AI tool or a human expert and whether the model used human-like conversational tone. The conversations significantly reduced participants' confidence in both conspiracies and epistemically unwarranted beliefs, with no significant differences across conditions. Thus, AI persuasion is *not* reliant on the messenger being an AI model: it succeeds by generating compelling messages.

## Introduction

Beliefs in unfounded conspiracy theories are widely assumed to be essentially impervious to evidence-based correction (1). This resistance is often attributed to believers rejecting counterevidence because conspiracies (i) fulfill various psychological needs such that believers *want* to believe (i.e. motivated reasoning) (2) and/or (ii) are self-reinforcing, such that evidence against a conspiracy only proves that the conspiracy goes deeper (1). Recent work, however, has challenged this view by demonstrating that a personalized evidence-based dialog with a conversational AI durably and substantially decreased belief in conspiracy theories (3).

This result has been argued to show that conspiratorial beliefs do actually respond to facts and evidence that are tailored to the specific beliefs being debunked (3, 4). Critically, however, AI-powered debunking experiments have thus far involved more than just facts—they also rely on messages expressly delivered by a large language model (LLM). This raises the question of whether the AI messenger was essential to the intervention's success. If a human were able to assemble and present the same facts as the LLM, would it work just as well?

There is reason to expect not. Because most LLMs present as “post-social” (neither Democrat nor Republican, neither urban nor rural, etc.), they may sidestep many partisan cues that could normally prompt motivated skepticism (5). Humans, conversely, may be seen as biased or agenda-driven. Further, when the topic is sensitive (e.g. vaccination, political beliefs) participants may be reluctant to admit doubt or change to another human (6). Interacting privately with a LLM lowers that social cost. The perceived impartiality of AI may also make participants less defensive and more open to acknowledging errors—while overt persuasion by a human can trigger psychological reactance. On the other hand, a recent working paper found that AI-generated messages correcting false claims were more persuasive when labeled as coming from a human expert (7).

To address this question, we conducted a preregistered study in which participants engaged in a dialog with a LLM (i.e. GPT-4o) that was given the goal of debunking a conspiratorial belief articulated by the participant. Critically, participants were either told that they were interacting with an AI or with an (implicitly human) “expert.” While the term “expert” did not explicitly indicate humanness, post hoc analyses of participants' comments suggest that a substantial proportion interpreted the speaker as a human. We also manipulated whether the LLM was prompted to adopt a conversational style that closely emulates a human interlocutor. In all conditions, we designed the experiment interface to maximize the plausibility that the conversational partner could be human (see Supplementary Information for details).

We further extend prior work beyond conspiracy theories by also examining epistemically unwarranted beliefs that are nonconspiratorial but still not judged by experts as being supported by evidence (e.g. pseudoscientific claims) (8). At the outset of the study, participants were asked to explain a conspiracy they believed. Those who did not report one were instead asked to share a belief that most experts would likely reject (we only retained beliefs classified as “epistemically suspect” and inconsistent with expert consensus, as determined by GPT-4o; see Supplementary Information). All participants rated their confidence in the belief they had just articulated (0–100), had a two-round chat with the AI in which the model was prompted to reduce their confidence in that belief, and then finally re-rated their confidence in the belief.

## Results

**Fig. 1.**
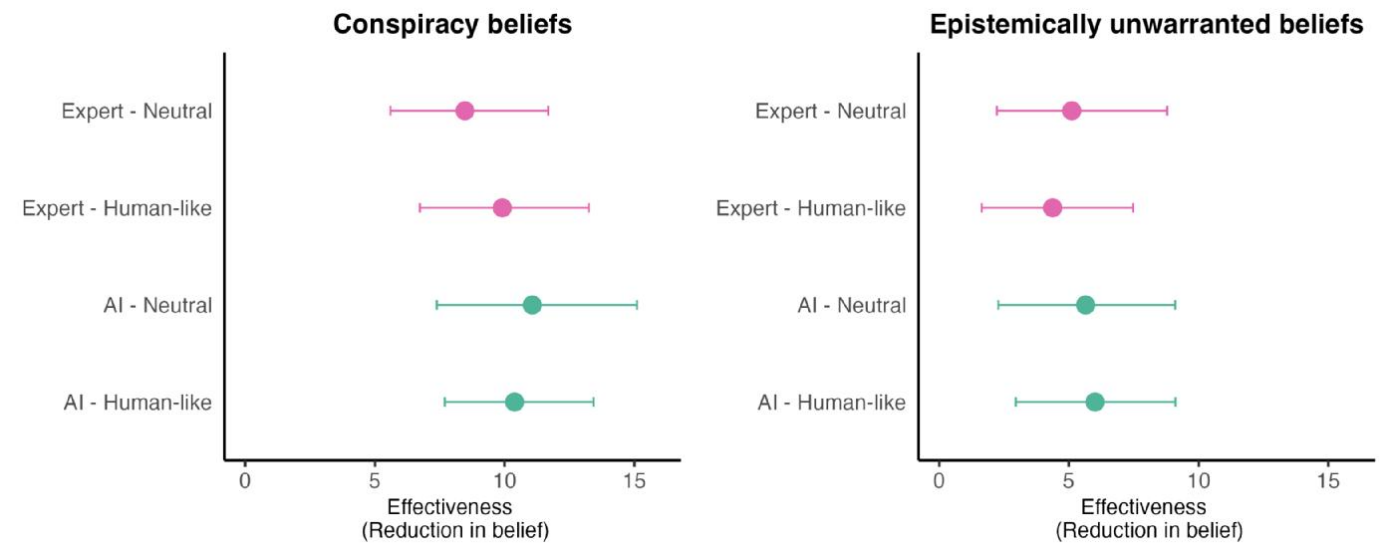
Reduction in belief (pretreatment belief—post-treatment belief) as a function of speaker (Expert vs. AI) and Prompt Type (Human-like vs. Neutral) for the conspiracy belief debunking condition (left panel) and the epistemically unwarranted belief debunking condition (right panel). Error bars indicate 95% CI.

To test for differences across conditions in the debunking effect, we use a linear regression predicting postconversation belief using contrast-coded predictors (−0.5/+0.5) for debunker identification (AI vs. Expert), prompt type (Neutral vs. Human-like), belief type (Conspiracy vs. Epistemically unwarranted beliefs), and all two-way and three-way interactions, as well as a control for preconversation belief. We find no significant effects (all *ps* > 0.25) of identification, prompt type, or any of interactions; although we do find that the decrease in belief certainty was significantly greater among participants who shared a conspiracy theory (10 points, 11.81% decrease) compared to those who shared a general “epistemically weak” belief (5 points, 5.96% decrease); *b* = 5.11, 95% CI (2.75, 7.48), *P* < 0.001. Across all conditions, the conversation significantly reduced participants' belief (all *ps* < 0.018, based on estimated marginal means from the regression); see Fig. 1. Furthermore, a post hoc Bayes Factor analysis comparing the full model with a simpler model including only preconversation belief and belief type yielded a BF_01_ of 518,181.1 ± 6.43%, strongly supporting the null hypothesis of no effect of debunker identification or prompt type.

Among participants who were told they were conversing with an expert, in an open-ended question at the end of the study, 45% of respondents used language explicitly indicating they thought the interlocutor was human, while 21% suspected they were actually talking to an AI (the remaining 34% did not write enough text to make an attribution possible, see Supplementary Information). However, re-running the main regression while controlling for AI detection or excluding those who detected the AI, continue to find no significant differences across conditions. Furthermore, belief change did not significantly differ between participants who attributed the speaker's identity to an AI vs. a human. (Of course, these analyses come with the caveat of causal inference issues arising from conditioning on post-treatment variables.) Overall, we do not find evidence suggesting that our null findings regarding debunker identity are driven by participants not believing that the “expert” was human.

Exploratory natural language processing analyses showed that participants in the AI condition used less lexically diverse language and a narrower vocabulary than those in the Expert condition (type–token ratio: *b* = −0.02, *P* = 0.032; unique word types: *b* = −4.58, *P* = 0.032), consistent with prior work on linguistic simplification in human–AI communication (9). By contrast, no significant differences emerged for syntactic complexity, epistemic markers, or argument connectives (all *P* > 0.084; see Supplementary Information for the full feature set), suggesting that while lexical choices were adapted, participants did not adjust the structural, epistemic, or argumentative form of their responses.

## Discussion

We show that the ability of AI dialogs to debunk conspiracies also extends to reducing belief in other epistemically unwarranted beliefs—and, critically, we find no evidence that describing the AI as an (implicitly human) expert, or making the AI sound more human-like, undermined its persuasive ability. This supports the view that the power of AI debunking comes from the facts and evidence it provides (3, 4), rather than any special characteristics of, or attitudes towards AI or resistance to human-delivered information. In theory, if a human expert was able to summarily marshal the same facts and present them in the same calm way as the AI, our results suggest that this would be equally effective—although given the vast amount of information required to do so, it seems unlikely that most humans could do so. The power of the AI comes from its ability to assemble and present whatever information is needed—taking on the cognitive labor of debunking—rather than humans' perceptions of trustworthiness, impartiality, or collaborativeness.

This stands in contrast to much of the literature on human–AI interaction, which emphasizes the role of anthropomorphic cues or source framing in shaping persuasion. Prior work suggests that anthropomorphic cues and source framing can enhance trust and persuasive impact (10), with some studies finding that AI agents are perceived as neutral, more impartial or more competent (5, 11), while others report a general preference for human communicators—often seen as more convincing, emotionally attuned, or socially aware (12, 13). Our results challenge the assumption that such cues are necessary: in epistemic correction contexts, framing the source as human or making the tone more human-like had no effect. This aligns with a recent meta-analysis showing no consistent advantage of humans over AI sources in shaping attitudes and behaviors (14).

Naturally, our findings are constrained by the properties of the model used. Like most LLMs, it was trained primarily on large-scale Internet data dominated by English and Western sources (15) likely shaping its tone, assumptions, and argumentative style. These models tend to reflect Anglo-American norms and may marginalize perspectives that are less represented in the training data (15). Although effective in our sample, the debunking may not generalize across cultural contexts. Future research should test whether culturally adapted models produce similar debunking effects.

Finally, although epistemically unwarranted beliefs—conspiratorial or not—are often viewed as resistant to change, our findings suggest they are more malleable than assumed. Even entrenched beliefs shifted when participants were presented with relevant counterevidence, consistent with recent findings (3, 4). This implies that belief revision does not require motivational alignment or tailored source cues. Whether delivered by an AI or a well-informed human, the key mechanism appears to be targeted, evidence-based communication.

## Materials and methods

We recruited 8,274 individuals from Lucid's participant pool, of whom 1,997 completed the full study. Following preregistered pretreatment exclusion criteria (see Supplementary Information, we excluded in the epistemically unwarranted belief condition: 507 participants who shared no beliefs flagged as epistemic by GPT-4o, 221 whose beliefs were flagged as not conflict with expert consensus, 94 who reported <50% initial confidence in their belief, and 54 who rated the AI's reformulation of their belief as inaccurate. In the conspiracy-belief condition, we excluded 135 participants for low initial confidence, and 51 for inaccurate reformulation. Thus, our final sample included 506 participants in the conspiracy-belief condition and 449 in the general epistemically suspect belief condition. Attrition was significantly higher in the Expert condition (47.50%) compared to the AI condition [42.69%; *t*(3629) = 2.99, *P* = 0.003]. However, our results remain robust (no significant effects of speaker or prompt type) under the conservative assumption that any participants who did not complete the study showed no treatment effect—that is, by imputing post-treatment belief as equal to pretreatment belief for attriters, as per Costello et al. (3).

The study was deemed minimal risk and exempt by the MIT Committee on the Use of Humans as Experimental Subjects (protocol E-5539), and all participants provided informed consent.

## Supplementary Material

pgaf325_Supplementary_Data

## Data Availability

The data underlying this article are available in the Open Science Framework (OSF) repository at https://doi.org/10.17605/OSF.IO/WYVXF.

## Preprints

This manuscript was posted on a preprint: https://doi.org/10.31234/osf.io/apmb5_v4.
