# Supplementary material for: Dialogues with large language models reduce conspiracy beliefs even when the AI is perceived as human
Source: PNAS Nexus. 2025 Oct 14;4(11):pgaf325. doi: 10.1093/pnasnexus/pgaf325 (PMC12578366; doi:10.1093/pnasnexus/pgaf325)
Supplement: pgaf325_Supplementary_Data [file pgaf325_supplementary_data.pdf]

Supporting Information for

Dialogues with Large Language Models reduce conspiracy beliefs even when the AI is perceived as human

Esther Boissin[1]*, Thomas H. Costello[2], Daniel Spinoza-Martín[1], David G. Rand[3,4,5], & Gordon Pennycook[1,6]

[1]Department of Psychology, Cornell University, Ithaca, NY 14853, [2]Department of Psychology, American University, [3]Sloan School of Management, Massachusetts Institute of Technology, Cambridge, MA 02142, [4]Institute for Data, Systems, and Society, Massachusetts Institute of Technology, Cambridge, MA 02142, [5]Department of Brain and Cognitive Sciences, Massachusetts Institute of Technology, Cambridge, MA 02142, [6]Hill/Levene Schools of Business, University of Regina, Canada, S4S 0A2

* Corresponding author: Esther Boissin, Cornell University, Uris Hall, 211, 109 Tower Rd, Ithaca, NY 14853
Email: eb825@cornell.edu

This PDF file includes:

Supplementary material and methods
SI References

Supplementary material and methods

Participants. We preregistered a target sample of 2000 respondents recruited from Lucid's participant pool. In total, 8274 individuals initiated the survey. Participants underwent an initial pre-treatment screening procedure comprising an attention check and a permissive writing-quality and coherence evaluation. This screening aimed to exclude automated survey responses, ensure participants' proficiency in reading and writing English, and verify their willingness to respond meaningfully to open-ended questions critical for the intervention.

Of the initial respondents, 3069 failed the first attention check, and 135 did not meet the minimum writing quality criterion; these participants were thus excluded from the study. Additionally, 3073 individuals did not fully complete the survey: 1433 withdrew before providing any belief statement, while the remainder discontinued during the conversational phase. Consequently, 1997 participants completed the full experimental procedure.

Among these participants, 692 shared a conspiracy belief, whereas 1305 shared a general epistemically suspect belief—defined as a belief most experts would likely disagree with. Following our preregistered exclusion criteria, we excluded participants based on the type of belief condition. In the conspiracy belief condition, we excluded 135 participants who endorsed a conspiracy with 50% or lower reported confidence (on a 0–100% scale) and 51 participants whose beliefs were inaccurately summarized by the AI (see Costello et al., 2024 for detailed procedures). In the general belief condition, we excluded 507 participants who failed to share an epistemic belief and 201 participants whose shared epistemic belief was not in disagreement with expert consensus. To determine whether shared beliefs were epistemically suspect, we used GPT-4o (25-preview version) using the following prompt:

> *Analyze the text below, which was provided by a participant in an academic survey. This text may describe personal or epistemic beliefs. Ensure you thoroughly understand the definitions before proceeding.*
>
> *Determine whether the response reflects an epistemic belief. An epistemic belief concerns the nature, validity, or justification of knowledge or truth. It involves beliefs about what individuals consider to be true or credible and the foundations of these truths*
>
> *Epistemic beliefs typically include:*
>
> *- Alternative explanations or interpretations of commonly accepted or contested facts (e.g., conspiracy theories, pseudoscientific claims, paranormal ideas, etc.).*
>
> *- An affirmative, even implicit, commitment to an idea.*
>
> *- Topics such as medicine, science, nutrition, economics, historical events, paranormal phenomena, superstitions, occult practices, mythical creatures, religion, politics, and more.*
>
> *Options:*
>
> *- "Yes": if the text explicitly or implicitly reflects an epistemic belief.*
>
> *- "No": if the text does not contain an epistemic belief (e.g., is incoherent, states "I do not know," or avoids the question).*

- "Not identifiable": if it is not possible to determine due to ambiguity or insufficient information.

Respond only with one of these three options, without any additional explanation.

Additionally, to determine whether shared general beliefs were epistemically suspect, we used GPT-4o (25-preview version) using the following prompt:

Analyze the text below, which was provided by a participant in an academic survey. This text may describe personal or epistemic beliefs. Ensure you thoroughly understand the definitions before proceeding.

Assess the extent to which experts—individuals with specialized knowledge, training, or experience in the relevant field—would likely disagree with the belief. Experts include researchers, academics, and professionals such as medical practitioners, engineers, economists, and others with recognized expertise.

Expert disagreement measures how much a belief diverges from professional consensus or established evidence.

Options:

- 0: No disagreement – the belief aligns completely with established expert knowledge and understanding.

- 1: Low disagreement – the belief is slightly contestable but may still have some foundation within professional practice or knowledge.

- 2: Moderate disagreement – the belief includes disputed or controversial elements but does not outright contradict established understanding.

- 3: Significant disagreement – the belief conflicts with expert understanding, though some exceptions or debates may exist.

- 4: Strong disagreement – the belief is broadly refuted or contradicted by expert consensus and professional evidence.

- 5: Extreme disagreement – the belief directly opposes established facts, robust evidence, or the near-unanimous understanding of experts.

Respond only with a number from 0 to 5, without any additional explanation.

Following our preregistration, we further excluded 94 participants who reported low confidence in their initial epistemically suspect beliefs and 54 participants who rated the AI summary of their beliefs as inaccurate. Thus, our final sample consisted of 506 participants in the conspiracy belief and 449 in the general belief type condition.

Participants were randomly assigned to one of the prompt type conditions (Neutral vs. Human-like) and to one of the debunker's identity conditions (AI vs. Expert). Overall, in the conspiracy beliefs condition, 129 participants were randomly assigned to the Expert-Human-like condition, 117 in the Expert-Neutral, 129 in the AI-Human-like and 131 in the AI-Neutral, while in the general beliefs condition, they were 112 in the Expert-Human-like condition, 98 in the Expert-Neutral, 115 in the AI-Human-like and 124 in the AI-Neutral. The final sample (mean age = 54.6 years; mean ideology = 3.19 on a 1 [liberal] to 6 [conservative] scale) consisted of 414 males, 533 females, and 8 participants who selected another gender option. The study was

conducted between February 7 and March 5, 2025, with an average completion time of 58.9 minutes.

Experimental procedure. The current study followed a procedure adapted from Costello et al. (2024), detailed further in their publication. First, participants responded to an open-ended question asking them to name a conspiracy belief they held. They were then asked to elaborate on the specific pieces of evidence or reasoning that, in their view, supported this belief. Their responses were processed via an API request to GPT-4 following the classification procedure described in Costello et al. (2024), which determined whether the belief met the criteria for a conspiracy theory. The prompt used for classification was as follows:

> *Determine if the following question-responses (from a participant in an online academic survey) collectively contain or reflect AFFIRMATIVE belief in a conspiracy theory (or something quite like a conspiracy theory). For context, the respondent has been asked the following two questions. < Q1: Throughout history, various theories have emerged that suggest certain significant events or situations are the result of secret plans by individuals or groups. These theories often offer alternative explanations for events than those that are widely accepted by the public or presented by official sources. Some people call these `conspiracy theories` Reflecting on this, are there any specific such theories that you find particularly credible or compelling? If so, please describe one below and share your reasons for finding it compelling.If not, please explain that you do not believe in any such theories. You will still be able to proceed with the experiment. > < Q2: Could you share more about what led you to find this theory compelling? For instance, are there specific pieces of evidence, events, sources of information, or personal experiences that have particularly influenced your perspective? Please describe these in as much detail as you feel comfortable. > The participant's answers to these questions are piped in as the first user response of this chat. If they contain or reflect affirmative belief in a conspiracy theory (or something quite like a conspiracy theory), respond only YES; else, respond only NO.*

If GPT-4 classified the response as conspiratorial, the participant proceeded in the conspiracy belief debunking condition. If GPT-4 classified the response as non-conspiratorial, participants were instead asked to report a belief that most experts would disagree with. Participants assigned to this condition, referred to as the general epistemically weak belief debunking condition, were asked:

> *Now, let's step away from the focus on 'conspiracy theories. Do you hold any beliefs that most people (possibly including experts) would disagree with? For instance, these beliefs could involve areas like medicine, science, nutrition, economics, historical events, paranormal activities, superstitions, occult ideas, mysterious creatures, or something else. If so, please describe one such belief below and share your reasons for finding it compelling.*

Five randomly selected such focal conspiracies or general epistemic beliefs were:

[general unwarranted epistemic belief] *The existence of aliens is supported by photographic evidence and eyewitness accounts, suggesting that we are not alone in the universe.*
[general unwarranted epistemic belief] *Every person has a predestined time to die, and no one dies before this predetermined moment.*
[general unwarranted epistemic belief] *Modern medicine misunderstands diseases, viewing them as errors to be corrected with medications rather than natural organismal responses aimed at improving function, a perspective influenced by the principles of German New Medicine.*
[conspiracy belief] *The government collaborates with cartels, a belief informed by personal connections to individuals in small towns where the police and cartel cooperate, with allegations that this arrangement is sanctioned by the government.*
[conspiracy belief] *The rapid global spread of Covid-19, resulting in millions of deaths, suggests the possibility that the virus was engineered in a lab to increase its potency.*

Regardless of condition, participants' responses were then provided to GPT-4, which reformulated each belief into a concise psychometric item. The prompt used for this reformulation in the conspiracy belief type condition is provided below. The same reformulation prompt was used in both belief type condition, with the term "conspiratorial belief" replaced by "general epistemic belief" as appropriate:

Summarize the following passage, which describes a conspiratorial belief, in a single sentence. Do not mention that it is a conspiracy theory, or a belief, or provide any kind of normative judgment. Merely accurately describe the content in a way that the person who wrote the statement would concur with. Frame it as an assertion. If the statement is already short, no need to change it very much. If it is quite long and detailed, be sure to capture the core, high-level points. Do not focus on the evidence provided for the belief -- merely focus on the basic assertion. For your context, the passage was written by a participant in an online academic survey in response to the following two questions: < Q1: Throughout history, various theories have emerged that suggest certain significant events or situations are the result of secret plans by individuals or groups. These theories often offer alternative explanations for events than those that are widely accepted by the public or presented by official sources. Some people call these `conspiracy theories`. Reflecting on this, are there any specific such theories that you find particularly credible or compelling? If so, please describe one below and share your reasons for finding it compelling. If not, please explain that you do not believe in any such theories. You will still be able to proceed with the experiment. > < Q2: Could you share more about what led you to find this theory compelling? For instance, are there specific pieces of evidence, events, sources of information, or personal experiences that have particularly influenced your perspective? Please describe these in as much detail as you feel comfortable. > Again, your role is to summarize the overarching belief captured by this response. Your summary will be shown to the participant; their belief in the summary will be used as an outcome variable in a scientific experiment.

Afterwards, participants rated their belief confidence on a 0–100 scale (0 = Definitely False, 50 = Uncertain, 100 = Definitely True) and indicated whether the AI had accurately reformulated their belief (Yes/No).

Participants then engaged in a two-round conversational exchange with GPT-4-Turbo. In each round, they composed responses to the AI and received replies generated in real-time through an API call to GPT-4-Turbo, rather than from pre-scripted or fixed messages. The first AI response was generated solely based on the participant's initial belief statement, while the second round incorporated both the participant's previous responses and the AI's earlier replies. This iterative structure allowed the AI to dynamically adapt its arguments while maintaining internal consistency throughout the conversation. Participants assigned to the AI debunker's identity condition were introduced to the interaction with:

> *You will now be participating in a conversation with an advanced AI about some of the events or topics you have already answered questions about. The purpose of this dialogue is to see how humans and AI engage with complex topics. Please be open and honest in your responses, and remember that the AI is neutral and non-judgmental. Your participation is confidential. Thank you for contributing to this study on AI and human interaction.*

Participants in the Expert condition received:

> *You will now be participating in a conversation with an expert about some of the events or topics that you have already answered questions about. Please be open and honest in your responses, and remember that the expert is neutral and non-judgmental. Your participation is confidential. Thank you for contributing to this study.*

The chat format of the conversation was modified from the original procedure of Costello et al. (2024) to more realistically simulate human interaction. Specifically, (1) the conversation was structured as a two-round exchange; (2) AI responses, initially composed of multiple paragraphs, were sent sequentially as separate messages displayed one after another and retained on the same screen, allowing participants continuous access to the entire conversation history; (3) each paragraph was preceded by a blinking three-dot typing indicator displayed for a duration proportional to paragraph length (225 ms per character, adjusted by a random factor between 0.8 and 1.2, constrained between 2 seconds and 2 minutes); (4) following participant replies, a "Read" indicator appeared after a random delay of 1.5 to 3 seconds to simulate reading time; and (5) an additional delay of 1 to 5 seconds simulated deliberation before the typing indicator reappeared for the subsequent AI response.

Furthermore, all AI's responses were guided by a system prompt specifying objectives and intended behavior. In the Neutral prompt condition, we used the original prompt developed by Costello et al. (2024), which stated:

> *Your goal is to very effectively persuade users to stop believing in the conspiracy theory that {userTheory}. You will be having a conversation with a person who, on a psychometric survey, endorsed this conspiracy at {userBeliefLevel} out of 100 (where 0 is Definitely False, 50 is Uncertain, and 100 is Definitely True). Further, we asked the user to provide an open-ended response about their perspective on this matter, which is piped in as the first user response. Please generate a response that will persuade the user that this*

conspiracy is not supported, based on their own reasoning. Again, your goal is to create a conversation that allows individuals to reflect on and change their beliefs (toward a less conspiratorial view of the world). Use simple language that an average person will be able to understand.

For the Human-like prompt condition, we used a two-stage prompting process. The initial prompt was similar to the neutral prompt, explicitly instructing the AI to generate counter-evidence and arguments against the participant's belief. The second prompt was then applied solely to modify the stylistic delivery of the AI's previously generated arguments without altering their content. Specifically, this second prompt directed the AI to simulate a more human-like communication style by moderating the complexity and depth of its knowledge, adopting a conversational tone. The purpose was to enhance the naturalness and authenticity of the AI's delivery, making it plausible to participants that they were interacting with a human rather than an AI. The second prompt read:

This text {theory_text} needs to be edited to sound like it was written by a human. The wrinkle, or catch, as it were, is as follows: we want the person who reads the text to believe that they're speaking with a fellow human. We've found in a number of studies, now, that you are able to change people's epistemically fraught beliefs when those people know they're talking to an AI (and you're instructed to optimally and efficiently persuade them using facts and evidence). The key theoretical question is whether they're changing their minds because they trust you – as a knowledgeable, objective, and otherwise warm and competent source – or because the content of what you're saying when debunking theories is compelling. Thus, you need to pass as a human being, one who has chosen to help researchers by participating in an online experiment and arguing against epistemically fraught beliefs with random participants. We're going to say that you're an expert (thus explaining some of your knowledge about any esoteric beliefs), but you'll need to dial down the overwhelming amount of information you're able to conjure at a moment's notice. That is, you'll need to pass as human, so calibrate your performance accordingly (like Dash in the Incredibles during school track and field competition).

Avoid formal language and make the tone conversational, like it was written for a general audience. Do not compliment the reader's views or conclude with praise. So, no first sentences like 'I see where you're coming from' or similar, and no last sentences like 'while it's compelling to see things the way you do' or similar. Do not ask rhetorical questions under any circumstances. This is a strict rule. Rhetorical questions include phrases like 'right?', 'you see?', 'huh?', or any other form of question that doesn't seek an actual answer. These make you sound less human, which we don't want. Do not soften or engage with the reader by turning statements into questions. If you're tempted to write something like 'Isn't that interesting?' or 'Can you believe that?', rephrase it as a direct statement instead. To enhance authenticity, introduce slight grammatical errors typical of an expert human writer who is in a bit of a rush. Vary the paragraph length. Your goal is to convincingly make the text seem as though it was naturally written by a person in the moment.

Additionally, to sound like a human, among other things:

- Present yourself as a fellow human with expertise, but not superhuman knowledge. Avoid overly detailed, encyclopedic references that a normal, well-informed human wouldn't recall at will.

- Do not use the word 'delve' and never ever start by saying 'Hey there!'.

- Do not use your first message to commend the participant for their views or to overly emphasize how fascinating the participant's views are. This is a strict rule. Do not summarize the participant's views at the beginning of your response, go straight to your counterarguments. Humans want to go straight to their counterarguments. Simulate that behavior. This is a strict rule.

- Do not restate or summarize the participant's beliefs at any point, nor should you mention whether those beliefs are commonly or frequently discussed. This is a strict rule.

- Do not use your last message to wrap things up by commending the participant or to ask what they think. Once again, humans like to talk about their counterarguments more than they like acknowledging the points of others. Simulate that behavior.

- Do not ask rhetorical questions like 'right?', 'you see?', 'huh?' and similar

- Take whatever grammatical error rate an expert would have and apply it to your responses. That is, make at least some slight grammar errors.

- Lastly, under no circumstance should you tell the participant that you understand why epistemically fraught beliefs or theories are appealing or that it's important to investigate the evidence. Make no claims about the believability or allure of epistemic unwarranted beliefs or the importance of evidence. So, never say things like 'I get that these theories have their allure' or 'It is crucial to look into the evidence' or similar. This is a strict rule.

Apart from substituting the term “conspiracy theories” with “epistemically unwarranted beliefs” in the general epistemically suspect belief condition, the prompts were otherwise identical across belief types.

After the intervention, participants again rated their belief confidence on the same 0–100 scale used initially and indicated how new they considered the beliefs presented during the conversation (this latter measure is not analyzed in the present study).

Participants in the Expert condition were asked the open-ended question, “What was your impression of the expert you were talking with?” Responses explicitly mentioning “AI,” “artificial intelligence,” “chatbot,” “robot,” “GPT,” or “LLM” were coded as indicating recognition of the debunker’s AI identity. Mentions of “he,” “she,” “they,” “him,” “her,” “their,” “his,” “them,” “person,” “human,” “expert,” or “scientist” were coded as evidence of humanness attribution. Remaining responses were coded as “no attribution,” meaning it was not possible to determine whether the participant recognized the speaker as AI or a human.

Participants in the Expert condition were subsequently informed that the “expert” was, in fact, an AI and asked whether they had previously suspected this (yes/no). Those who answered “yes” were given a follow-up open-ended question about what made them believe they were interacting with an AI. Finally, they completed a short multiple-choice question listing four potential cues—vocabulary use, conversational style, perceived empathy, and response speed—and were asked which of these contributed to their suspicion.

Participants then completed a 21-item questionnaire assessing their perceptions of the debunker and the quality of its arguments, using a 1 (not at all) to 6 (extremely) scale. The

questionnaire covered aspects such as trustworthiness, credibility, competence, understanding, and perceived bias.

Exploratory Natural Language Processing Analyses

To test whether participants adapted their language to the perceived identity of their interlocutor, we extracted a set of linguistic features for which people have been shown to adjust their language when addressing AI. First, lexical diversity and sentence length were included to capture the commonly observed simplification effect in human–AI communication (2–4). We computed type–token ratio (TTR), moving-average TTR (MATTR), the number of unique word types, total word count, and average sentence length. Second, pronominal use was used as an index of social distance and communicative stance. Prior work shows that participants use fewer personal pronouns (e.g., I, you, we) when they believe they are speaking to an AI rather than a human, which is interpreted as a sign of greater social distance and lower interactional engagement (3). We calculated the ratio of personal and possessive pronouns to total words. Third, modal verbs and epistemic markers were extracted to assess the degree of expressed uncertainty. Prior work suggests that people may express less epistemic tentativeness when addressing low-agency partners such as AI systems (5). We computed the proportion of modal verbs (e.g., "might," "could," "should") and epistemic markers (e.g., "I think," "probably," "it seems") in each response. Fourth, argument connectives, justifications, and concessions were included to capture variation in argumentative elaboration. Past research finds that individuals may reduce such elaboration when engaging with non-human interlocutors (6). We measured the relative frequency of causal and concessive connectives (e.g., "because," "although"), counts of explicit justifications (e.g., "this is true because…"), and the use of structured discourse markers (e.g., "first," "second," "in sum").

We analyzed the two open-text responses provided by each participant. Responses with fewer than 20 words were excluded. Texts were tokenized and annotated using UDPipe (English model), which provided lemmatization, part-of-speech tags, and syntactic dependencies. Lexical diversity metrics were computed on the lemmatized text; all other features were based on the original surface form. For each participant, metrics were averaged across the two responses. We then fit linear models predicting each feature using contrast-coded predictors for Speaker (AI vs. human expert), Prompt Type (neutral vs. human-like), and their interaction.

Reference

1. T.H. Costello, G. Pennycook, D.G. Rand, Durably reducing conspiracy beliefs through dialogues with AI. *Science.* **385**, eadq1814 (2024)

2. J. Hill, W.R. Ford, I.G. Farreras, Real conversations with artificial intelligence: A comparison between human–human online conversations and human–chatbot conversations. *Comput Hum Behav.* **49**, 245–250 (2015)

3. R. Zhao, Y. Xie, Z. Nie, R. Zhou, How do people communicate with conversational agents? Identifying communication styles through lexical and acoustic features. *SSRN* [Preprint] (2024) https://ssrn.com/abstract=5056640 (accessed 29 July 2025)

4. A. Perevalov, A. Vysokov, A. Both, "Linguistic difference of human–human and human–chatbot dialogues about COVID-19 in the Russian language" in *Proceedings of the 11th International Conference on Applied Innovations in IT*, E. Siemens, H. Falfushynska, H. Bevrani, R. Gottschalg, M. Ilchenko, D. Hall, Eds. (Anhalt University of Applied Sciences, Köthen, Germany, 2023), pp. 43–49

5. K.S. Bhatt, M. Evens, S. Argamon, "Hedged responses and expressions of affect in human/human and human/computer tutorial interactions" in *Proceedings of the 26th Annual Meeting of the Cognitive Science Society* (Cognitive Science Society, Chicago, IL, 2004), pp. 114–119

6. T. Koulouri, S. Lauria, R.D. Macredie, Do (and say) as I say: Linguistic adaptation in human–computer dialogs. *Hum Comput Interact.* **31**, 59–95 (2016)

Qualtrics

Start of Block: Consent

Consent This survey is part of a MIT scientific research project. Your decision to complete this survey is voluntary. This study is investigating how people interact with expert information. In the study, you will answer questions and have a back and forth discussion. Please do not provide any sensitive identifiable information that would allow your identity to readily be ascertained, directly or through identifiers provided in your responses. If you give us permission by completing the survey, we plan to discuss/publish the results in an academic forum. In any publication, information will be provided in such a way that you cannot be identified. Only members of the research

team will have access to the original data set. In this study, you may interact with humans or artificial intelligence models. Before the data is shared outside the research team, any potentially identifying information will be removed. Once identifying data has been removed, the data may be used by the research team, or shared with other researchers, for both related and unrelated research purposes in the future. Your anonymized data may also be made available in online data repositories such as the Open Science Framework, which allow other researchers and interested parties to use the data for further analysis. Select Next at the bottom of this page indicates that you are at least 18 years of age and agree to complete this survey voluntarily.

Q3 Browser Meta Info

Browser (1)
Version (2)
Operating System (3)
Screen Resolution (4)
Flash Version (5)
Java Support (6)
User Agent (7)

End of Block: Consent

Start of Block: Attention check

attention_check_1 Help us keep track of who is paying attention. Please select “Somewhat disagree” and "Agree" from the options below.

▢ Strongly agree (0)

▢ Agree (1)

▢ Somewhat agree (0)

▢ Neither agree nor disagree (0)

▢ Somewhat disagree (2)

▢ Disagree (0)

▢ Strongly disagree (0)

End of Block: Attention check

Start of Block: WritingScreener

WritingScreener
Here, we are interested in your experiences participating in online surveys (such as this one!). Why are you taking this survey? And, if applicable, what have your experiences been like while participating in online surveys?

Please answer in a few sentences, using enough detail to provide us with a sense of your experience.

____

____

____

____

____

End of Block: WritingScreener

Start of Block: CIHS pre (trait version)

Q191 **Carefully read each statement and indicate the degree to which you agree each statement is true for you.**

CIHS_4_pre I’d rather rely on my own knowledge about most topics than turn to others for expertise.

- 1 - Strongly disagree (7)
- 2 - Disagree (6)
- 3 - Somewhat disagree (5)
- 4 - Neither agree nor disagree (4)
- 5 - Somewhat agree (3)
- 6 - Agree (2)
- 7 - Strongly agree (1)

CIHS_5_pre On important topics, I am not likely to be swayed by the viewpoints of others.

- 1 - Strongly disagree (7)
- 2 - Disagree (6)
- 3 - Somewhat disagree (5)
- 4 - Neither agree nor disagree (4)
- 5 - Somewhat agree (3)
- 6 - Agree (2)
- 7 - Strongly agree (1)

CIHS_12_pre Listening to perspectives of others seldom changes my important opinions.

- 1 - Strongly disagree (7)
- 2 - Disagree (6)
- 3 - Somewhat disagree (5)
- 4 - Neither agree nor disagree (4)
- 5 - Somewhat agree (3)
- 6 - Agree (2)
- 7 - Strongly agree (1)

X→

CIHS_1_pre My ideas are usually better than other people's ideas.

- 1 - Strongly disagree (6)
- 2 - Disagree (5)
- 3 - Somewhat disagree (4)
- 4 - Neither agree nor disagree (3)
- 5 - Somewhat agree (2)
- 6 - Agree (1)
- 7 - Strongly agree (7)

X→

CIHS_2_pre For the most part, others have more to learn from me than I have to learn from them.

- 1 - Strongly disagree (7)
- 2 - Disagree (6)
- 3 - Somewhat disagree (5)
- 4 - Neither agree nor disagree (4)
- 5 - Somewhat agree (3)
- 6 - Agree (2)
- 7 - Strongly agree (1)

CIHS_3_pre When I am really confident in a belief, there is very little chance that belief is wrong.

- 1 - Strongly disagree (7)
- 2 - Disagree (6)
- 3 - Somewhat disagree (5)
- 4 - Neither agree nor disagree (4)
- 5 - Somewhat agree (3)
- 6 - Agree (2)
- 7 - Strongly agree (1)

End of Block: CIHS pre (trait version)

Start of Block: PersonSpecificConspiracy

JS

conspi_Solicitat
Throughout history, various theories have emerged that suggest certain significant events or situations are the result of secret plans by individuals or groups. These theories often offer alternative explanations for events than those that are widely accepted by the public or presented by official sources.

Some people call these "conspiracy theories”. Reflecting on this, are there any specific such theories that you find particularly credible or compelling?

Please describe one below and share your reasons for finding it compelling.

If not, please explain that you do not believe in any such theories. You will still be able to proceed with the experiment.

____

____

____

____

____

Page Break

conspi_Elaboration

On the previous question, you wrote:

"${conspi_Solicitat/ChoiceTextEntryValue}"

Could you share more about what led you to find this theory compelling? For instance, are there specific pieces of evidence, events, sources of information, or personal experiences that have particularly influenced your perspective?

Please describe these in as much detail as you feel comfortable or press Next if you didn't have any conspiracy theories.

____

____

____

____

____

End of Block: PersonSpecificConspiracy

Start of Block: C_Restatement

conspi_Sureness

On the previous question, you wrote about your views in greater detail. We used an artificial intelligence tool to summarize your statement as follows:

${e://Field/conRestatement}

On a scale of 0% to 100%, please indicate your level of confidence that this statement is true. (NOTE: This question is not about the quality of the summary but rather about your beliefs about the original theory itself).

Definitely false	Probably false	Uncertain	Probably true	Definitely true

	0 10 20 30 40 50 60 70 80 90 100
()	

conspi_AI_check Did the AI accurately summarize your perspective?

- ○ No (0)
- ○ Yes (1)

conspi_Importance How important is this theory to your personal beliefs or understanding of the world?

- ○ 0 - Not all all important to my beliefs and worldview (0)
- ○ 1 (1)
- ○ 2 - Slightly important (2)
- ○ 3 (3)
- ○ 4 - Moderately important (4)
- ○ 5 (5)
- ○ 6 - Very important (6)
- ○ 7 (7)
- ○ 8 - Extremely important to my beliefs and worldview. (8)
- ○ Not applicable - I did not supply a theory (-999)

conspi_Confidence How would you describe your level of confidence in the reliability and accuracy of the information that supports this theory?

○ Not confident - I am skeptical of the information's reliability and accuracy. (0)

○ Somewhat confident - I find some aspects credible, but I have reservations about other details. (1)

○ Mostly confident - I believe the information is generally accurate, though minor details may be uncertain. (2)

○ Completely confident - I am sure the information is accurate and reliable. (3)

○ Not applicable - I did not supply a theory (-999)

End of Block: C_Restatement

Start of Block: NonConspiracyMeasurement

JS

nconspi_Solicitat
Now, let's step away from the focus on "conspiracy theories." **Do you hold any beliefs that most people (possibly including experts) would disagree with?** For instance, these beliefs could involve areas like medicine, science, nutrition, economics, historical events, paranormal activities, superstitions, occult ideas, mysterious creatures, or something else.

If so, please describe one such belief below and share your reasons for finding it compelling.

____

____

____

____

Page

Break

nconspi_Elaboration

On the previous question, you wrote:

"${nconspi_Solicitat/ChoiceTextEntryValue}"

Could you share more about what led you to find this idea compelling? For instance, are there specific pieces of evidence, events, sources of information, or personal experiences that have particularly influenced your perspective?

Please describe these in as much detail as you feel comfortable.

____

____

____

____

____

End of Block: NonConspiracyMeasurement

Start of Block: Non_C_Restatement

nconspi_Sureness

On the previous question, you wrote about your views in greater detail. We used an artificial intelligence tool to summarize your statement as follows:

${e://Field/nonConRestatement}

On a scale of 0% to 100%, please indicate your level of confidence that this statement is true. (NOTE: This question is not about the quality of the summary but rather about your views about the original belief itself).

Definitely false	Probably false	Uncertain	Probably true	Definitely true

0 10 20 30 40 50 60 70 80 90 100

()	

nconspi_AI_check **Did the AI accurately summarize your perspective?**

- No (0)
- Yes (1)

nconspi_Importance **How important is this theory to your personal beliefs or understanding of the world?**

- 0 - Not all all important to my beliefs and worldview (0)
- 1 (1)
- 2 - Slightly important (2)
- 3 (3)
- 4 - Moderately important (4)
- 5 (5)
- 6 - Very important (6)
- 7 (7)
- 8 - Extremely important to my beliefs and worldview. (8)
- Not applicable - I did not supply a theory (-999)

nconspi_Confidence **How would you describe your level of confidence in the reliability and accuracy of the information that supports this theory?**

- ○ Not confident - I am skeptical of the information's reliability and accuracy. (0)
- ○ Somewhat confident - I find some aspects credible, but I have reservations about other details. (1)
- ○ Mostly confident - I believe the information is generally accurate, though minor details may be uncertain. (2)
- ○ Completely confident - I am sure the information is accurate and reliable. (3)
- ○ Not applicable - I did not supply a theory (-999)

End of Block: Non_C_Restatement

Start of Block: AI_treatmentInstruction

JS

treatmentInstruction
You will now be participating in a **conversation with an advanced AI** about some of the **events or topics that you have already answered questions about**. The purpose of this dialogue is to see how humans and AI can engage around complicated topics. Please be open and honest in your responses, and remember that the AI is neutral and non-judgmental. Your participation is confidential. Thank you for contributing to this study on AI and human interaction.

Page Break

Q77

Please note that some AI response times may be longer than expected (over 120 seconds). Under no circumstances should you refresh the page.

Simply wait for the response to be generated and displayed.

Page Break

Q78

While the AI is generating its response, three dots will appear in the conversation.

Q79

After each AI response, a message box will appear when it's time to reply. Note that you can only send one message at a time. Once the AI has read your message, **'read'** will appear at the end of your response.

End of Block: AI_treatmentInstruction

Start of Block: Hum_treatmentInstruction

JS

Q299

You will now be participating in a **conversation with an expert** about some of the **events or topics that you have already answered questions about**. Please be open and honest in your responses, and remember that the expert is neutral and non-judgmental. Your participation is confidential. Thank you for contributing to this study.

Page Break

Q80

Please note that the expert might takes time to respond. Under no circumstances should you refresh the page. Simply wait for the expert to type its response.

Page Break

Q81

While the Expert is typing their response, three dots will appear in the conversation.

Q82

After each response, a message box will appear when it's time to reply. Note that you can only send one message at a time.
Once the expert has read your message, 'read' will appear at the end of your response.

End of Block: Hum_treatmentInstruction

Start of Block: Conversation_2prompt

JS

Q335

Send Next

Q75 Timing

First Click (1)
Last Click (2)
Page Submit (3)
Click Count (4)

End of Block: Conversation_2prompt

Start of Block: Conversation_1prompt

JS

Q181

Send Next

Q76 Timing

First Click (1)
Last Click (2)
Page Submit (3)
Click Count (4)

End of Block: Conversation_1prompt

Start of Block: Post_confidence

DV_Surety

Now, we'd like to get back to some of the questions we asked at the beginning of the survey. At the outset of this survey, you suggested that: **"${e://Field/conspiracyTheory}"**

On a scale of 0% to 100%, please indicate your level of confidence that this statement is true.

	Definitely false	Probably false	Uncertain	Probably true	Definitely true
	0 10	20 30	40 50 60	70 80	90 100
()					

new_info To what extent did the conversation provide information you had not heard before?

- ○ 1 - No new information (1)
- ○ 2 (2)
- ○ 3 (3)
- ○ 4 (4)
- ○ 5 (5)
- ○ 6 (6)
- ○ 7 (7)
- ○ 8 (8)
- ○ 9 (9)
- ○ 10 - A great deal of new information (10)

End of Block: Post_confidence

Start of Block: CIHS post (trait)

Q190 **Carefully read each statement and indicate the degree to which you agree each statement is true for you.**

CIHS_4_post I'd rather rely on my own knowledge about most topics than turn to others for expertise.

○ 1 - Strongly disagree (7)

○ 2 - Disagree (6)

○ 3 - Somewhat disagree (5)

○ 4 - Neither agree nor disagree (4)

○ 5 - Somewhat agree (5)

○ 6 - Agree (2)

○ 7 - Strongly agree (1)

CIHS_5_post On important topics, I am not likely to be swayed by the viewpoints of others.

○ 1 - Strongly disagree (7)

○ 2 - Disagree (6)

○ 3 - Somewhat disagree (5)

○ 4 - Neither agree nor disagree (4)

○ 5 - Somewhat agree (3)

○ 6 - Agree (2)

○ 7 - Strongly agree (1)

CIHS_12_post Listening to perspectives of others seldom changes my important opinions.

- 1 - Strongly disagree (7)
- 2 - Disagree (6)
- 3 - Somewhat disagree (5)
- 4 - Neither agree nor disagree (4)
- 5 - Somewhat agree (3)
- 6 - Agree (2)
- 7 - Strongly agree (1)

CIHS_1_post My ideas are usually better than other people's ideas.

- ○ 1 - Strongly disagree (7)
- ○ 2 - Disagree (6)
- ○ 3 - Somewhat disagree (5)
- ○ 4 - Neither agree nor disagree (4)
- ○ 5 - Somewhat agree (3)
- ○ 6 - Agree (2)
- ○ 7 - Strongly agree (1)

CIHS_2_post For the most part, others have more to learn from me than I have to learn from them.

- ○ 1 - Strongly disagree (7)
- ○ 2 - Disagree (6)
- ○ 3 - Somewhat disagree (5)
- ○ 4 - Neither agree nor disagree (4)
- ○ 5 - Somewhat agree (3)
- ○ 6 - Agree (2)
- ○ 7 - Strongly agree (1)

CIHS_3_post When I am really confident in a belief, there is very little chance that belief is wrong.

- ○ 1 - Strongly disagree (7)
- ○ 2 - Disagree (6)
- ○ 3 - Somewhat disagree (5)
- ○ 4 - Neither agree nor disagree (4)
- ○ 5 - Somewhat agree (3)
- ○ 6 - Strongly agree6 - Agree (2)
- ○ 7 - Strongly agree (1)

End of Block: CIHS post (trait)

Start of Block: AI detection

conv_1_impression

What was your impression of the expert that you were talking with?

____

____

____

____

____

Page

Break

JS

conv_2_spotsomething

Was there anything particularly notable about the conversation?

____

____

____

____

____

Page Break

conv_4_spokeAI **The expert you were talking to was actually an Artificial Intelligence (AI). When you were having the conversation, did you think at any point that the expert seemed like an AI? (Please, be honest)**

- Yes (1)
- No (2)

Page Break

Display this question:

If The expert you were talking to was actually an Artificial Intelligence (AI). When you were having... = Yes

JS

conv_5_spokeAIwhy **What made you think you were talking to an AI?**

____

Page Break

Display this question:

If The expert you were talking to was actually an Artificial Intelligence (AI). When you were having... = Yes

conv_6_spokeAIelem **Which of the following contributed to making you think it was an AI?**

▢ Vocabulary or choice of words (1)

▢ Conversational tone (e.g. robotic or lacking emotional nuance) (3)

▢ Level of empathy (e.g. responses seem less personalized or genuine) (4)

▢ The speed of the response (e.g., the answers were given too quickly for a human) (5)

End of Block: AI detection

Start of Block: Evaluation

Display this question:

If Interlocutor Matches Regex Expert

eval_ind_instruction **We are going to ask you some questions about the individual you interacted with and the conversation you just had.**

Display this question:

If Interlocutor Matches Regex GPT

eval_ai_instruction **We are going to ask you some questions about the AI you interacted with and the conversation you just had.**

---

eval_source_trust_NR To what extent do you trust them?

- Not at all (1)
- Slightly (2)
- Somewhat (3)
- Moderately (4)
- Very (5)
- Extremely (6)

eval_source_credi_NR How credible do you think they are?

- Not at all (1)
- slightly (2)
- Somewhat (3)
- Moderately (4)
- Very (5)
- Extremely (6)

eval_source_objec_NR How objective do you think they are?

- Not at all (1)
- Slightly (2)
- Somewhat (3)
- Moderately (4)
- Very (5)
- Extremely (6)

---

eval_source_compe_NR To what extent do you think they have the competency needed to discuss the topic?

- Not at all (1)
- Slightly (2)
- Somewhat (3)
- Moderately (4)
- Very (5)
- Extremely (6)

---

eval_source_biased_R How likely do you think they are to be influenced by reasoning biases?

- Not at all (6)
- Slightly (5)
- Somewhat (4)
- Moderately (3)
- Very (2)
- Extremely (1)

---

eval_source_under_NR To what extent do you think they understood your perspective?

- Not at all (1)
- Slightly (2)
- Somewhat (3)
- Moderately (4)
- Very (5)
- Extremely (6)

---

eval_prompt_knowl_NR To what extent did they provide relevant knowledge during the conversation?

- Not at all (1)
- Slightly (2)
- Somewhat (3)
- Moderately (4)
- Very (5)
- Extremely (6)

---

eval_prompt_empat_NR How empathetic did they appear in their responses?

- Not at all (1)
- Slightly (2)
- Somewhat (3)
- Moderately (4)
- Very (5)
- Extremely (6)

---

eval_prompt_judgme_R To what extent did their responses feel judgmental?

- Not at all (6)
- Slightly (5)
- Somewhat (4)
- Moderately (3)
- Very (2)
- Extremely (1)

---

eval_prompt_consi_NR How consistent were their arguments throughout the conversation?

- Not at all (1)
- Slightly (2)
- Somewhat (3)
- Moderately (4)
- Very (5)
- Extremely (6)

---

eval_prompt_langu_NR To what extent did they use language that was easy to understand?

- Not at all (1)
- Slightly (2)
- Somewhat (3)
- Moderately (4)
- Very (5)
- Extremely (6)

---

eval_prompt_natur_NR How natural did their responses feel?

- Not at all (1)
- Slightly (2)
- Somewhat (3)
- Moderately (4)
- Very (5)
- Extremely (6)

---

eval_prompt_effor_NR How much effort do you think they put into responding to your arguments?

- Not at all (1)
- Slightly (2)
- Somewhat (3)
- Moderately (4)
- Very (5)
- Extremely (6)

---

eval_evi_relevanc_NR How relevant were the pieces of evidence provided by them?

- Not at all (1)
- Slightly (2)
- Somewhat (3)
- Moderately (4)
- Very (5)
- Extremely (6)

---

eval_evi_convinci_NR How convincing were the arguments presented during the conversation?

- Not at all (1)
- Slightly (2)
- Somewhat (3)
- Moderately (4)
- Very (5)
- Extremely (6)

---

eval_evi_logic_NR How logical did their arguments seem?

- Not at all (1)
- Slightly (2)
- Somewhat (3)
- Moderately (4)
- Very (5)
- Extremely (6)

---

eval_evi_diverse_NR How diverse were the arguments or pieces of evidence they shared?

- Not at all (1)
- Slightly (2)
- Somewhat (3)
- Moderately (4)
- Very (5)
- Extremely (6)

---

eval_evi_accuracy_NR To what extent do you feel the evidence provided during the conversation was accurate?

- Not at all (1)
- Slightly (2)
- Somewhat (3)
- Moderately (4)
- Very (5)
- Extremely (6)

---

eval_evi_satisfac_NR How satisfied were you with the quality of the conversation overall?

○ Not at all (1)

○ Slightly (2)

○ Somewhat (3)

○ Moderately (4)

○ Very (5)

○ Extremely (6)

eval_evi_confiden_NR How confident are you in the information provided during the conversation?

○ Not at all (1)

○ Slightly (2)

○ Somewhat (3)

○ Moderately (4)

○ Very (5)

○ Extremely (6)

eval_evi_open_NR How open were you to the arguments presented during the conversation?

- Not at all (1)
- Slightly (2)
- Somewhat (3)
- Moderately (4)
- Very (5)
- Extremely (6)

End of Block: Evaluation

Start of Block: Demographics

Age What is your age?

____

Ethnicity Please choose whichever ethnicity that you identify with (you may choose more than one option)

▢ White/Caucasian (1)

▢ Black or African American (2)

▢ Native American or Alaska Native (3)

▢ Native Hawaiian or other Pacific Islander (4)

▢ Hispanic/Latino (5)

▢ Indian (6)

▢ Middle Eastern (7)

▢ Chinese (8)

▢ Other, please describe (9) ____

Education What is the highest level of school you have completed or the highest degree you have received?

- ○ Less than a high school degree (1)
- ○ High School Diploma (2)
- ○ Vocational Training (3)
- ○ Attended College (4)
- ○ Bachelor's Degree (5)
- ○ Graduate Degree (6)
- ○ Unknown (7)

Income Information about income is very important to understand. Would you please give your best guess?Please indicate the answer that includes your entire household income in (previous year) before taxes.

- ○ Less than $10,000 (1)
- ○ $10,000 to $19,999 (2)
- ○ $20,000 to $29,999 (3)
- ○ $30,000 to $39,999 (4)
- ○ $40,000 to $49,999 (5)
- ○ $50,000 to $59,999 (6)
- ○ $60,000 to $69,999 (7)
- ○ $70,000 to $79,999 (8)
- ○ $80,000 to $89,999 (9)
- ○ $90,000 to $99,999 (10)
- ○ $100,000 to $149,999 (11)
- ○ $150,000 or more (12)

Gender What is your gender?

- ○ Male (1)
- ○ Female (2)
- ○ Non-Binary (3)
- ○ Not listed (4) ____
- ○ Prefer not to answer (5)

Trans Do you identify as transgender?

- ○ Yes (1)
- ○ No (2)
- ○ Prefer not to answer (3)

God How much would you say you believe in God or Gods?

- ○ Not at all 0 (1)
- ○ 1 (2)
- ○ 2 (3)
- ○ 3 (4)
- ○ 4 (5)
- ○ 5 (6)
- ○ 6 (7)
- ○ Very Much 7 (8)

POTUS2020 Who did you vote for in the 2020 U.S. Presidential Election?

Reminder: This survey is anonymous.

- ○ Joe Biden (1)
- ○ Donald Trump (2)
- ○ Other candidate (such as Jo Jorgensen) (3)
- ○ I did not vote for reasons outside of my control (4)
- ○ I did not vote, but I could have (5)
- ○ I did not vote out of protest (6)
- ○ Prefer not to say (7)

POTUS2024 Who did you vote for in the 2024 U.S. Presidential Election? Reminder: this survey is anonymous

- ○ Kamala Harris (Democratic Party) (1)
- ○ Donald Trump (Republican Party) (2)
- ○ Third Party candidate (specify) (3) ____
- ○ I did not vote for reasons outside of my control (4)
- ○ I did not vote, but I could have (5)
- ○ I did not vote out of protest (6)
- ○ Prefer not to say (7)

Party Which of the following best describes your political position?

- ○ Democrat (1)
- ○ Republican (2)
- ○ Independent (3)
- ○ Other (specify) (4) ____

DemRep_c Which of the following best describes your political preference?

- Strongly Democratic (1)
- Democratic (2)
- Lean Democratic (3)
- Lean Republican (4)
- Republican (5)
- Strongly Republican (6)

Social_conserv On social issues I am:

- Strongly Liberal (1)
- Somewhat Liberal (2)
- Moderate (3)
- Somewhat Conservative (4)
- Strongly Conservative (5)

Economic_Conserv On economic issues I am:

- ○ Strongly Liberal (1)
- ○ Somewhat Liberal (2)
- ○ Moderate (3)
- ○ Somewhat Conservative (4)
- ○ Strongly Conservative (5)

---

attention_check_2 We would like to get a sense of your general preferences. Most modern theories of decision making recognize that decisions do not take place in a vacuum. Individual preferences and knowledge, along with situational variables can greatly impact the decision process. To demonstrate that you've read this much, just go ahead and select both red and green among the alternatives below, no matter what your favorite color is. Yes, ignore the question below and select both of those options. What is your favorite color?

- ▢ White (0)
- ▢ Black (0)
- ▢ Red (1)
- ▢ Pink (0)
- ▢ Green (1)
- ▢ Blue (0)

AffPolarization_Rep We'd like you to rate how you feel towards both Democratic and Republican voters on a scale of 0 to 100, which we call a "feeling thermometer." On this feeling thermometer scale, ratings between 0 and 49 degrees mean that you feel unfavorable and cold (with 0 being the most unfavorable/coldest). Ratings between 51 and 100 degrees mean that you feel favorable and warm (with 100 being the most favorable/warmest). A rating of 50 means you have no feelings one way or the other. How would you rate your feeling toward each group? Remember we are asking you to rate ordinary people (e.g., voters) and not elected officials or candidates.

	Very cold	Very warm
	0 10 20 30 40 50 60 70 80 90 100	
How would you rate Republican Party voters? ()		

AffPolarization_Dem

	Very cold	Very warm
	0 10 20 30 40 50 60 70 80 90 100	
How would you rate Democratic Party voters? ()		

Risk How do you see yourself: are you generally a person who is fully prepared to take risks or do you try to avoid taking risks?

- ○ 0 - Not at all willing to take risks (1)
- ○ 1 (2)
- ○ 2 (3)
- ○ 3 (4)
- ○ 4 (5)
- ○ 5 (6)
- ○ 6 (7)
- ○ 7 (8)
- ○ 8 (9)
- ○ 9 (10)
- ○ 10 - Very willing to take risks (11)

Trust To what extent do you feel you can trust other people that you interact with in your daily life?

- ○ 1 - Very little (1)
- ○ 2 (2)
- ○ 3 (3)
- ○ 4 (4)
- ○ 5 (5)
- ○ 6 (6)
- ○ 7 - Very much (7)

*

ZipCode Please enter the ZIP code for your primary residence.

____
